# A model for improving postgraduate medical education using the GMC survey

**DOI:** 10.1192/bjo.2021.434

**Published:** 2021-06-18

**Authors:** Martin Schmidt, Timothy Leung

**Affiliations:** 1 Surrey and Borders Partnership NHS Foundation Trust; 2 East London NHS Foundation Trust

## Abstract

**Aims:**

To investigate whether the General Medical Council (GMC) National Training Surveys (NTS) can be analysed to develop a plan of action that improves postgraduate training.

**Background:**

As part of its role in quality assurance of medical training, the GMC conducts an annual survey of trainers and trainees. The Doctors in training survey, part of the NTS, consists of 70 questions which are grouped into 18 indicators of quality. At Surrey and Borders Partnership NHS Foundation Trust, we were keen to use the comprehensive data in the NTS to improve training. We analysed each question to create a plan of action to improve the quality of training.

**Method:**

We used data from the online reporting tool to calculate the scores for each question in the 2018 NTS. Taking into account the impact of year-on-year changes in the content of the survey, we examined the score, change from 2017 to 2018, and difference between the score and indicator mean to identify poorly-performing questions. Other questions with clear potential for further improvement were also highlighted. A plan of action was produced by the Leadership and Education Fellow and Director of Medical Education.

**Result:**

29 actions were identified. The most common were to ensure that information (e.g. job descriptions, professional opportunities) was accessible to trainees (8 actions); liaise with other teams (e.g. Human Resources, Safety team) (6); discuss issues with or provide information to trainers (5); discuss with trainees to contextualise survey results within their experiences (4); and ensure that information was delivered at induction (3).

To implement these actions, we conducted a workshop for trainers and held feedback meetings with trainees. 76.5% of trainers (13/17) and 88.5% of trainees (23/26) surveyed following these respective events agreed or strongly agreed that the NTS can be used to improve the training experience. A presentation on making the most of the placement was added to trainee induction and was rated excellent or good by all respondents (28/28). Posters were also produced to disseminate information. In the subsequent NTS, there was an improvement in SABP's performance in 12/18 indicators in the Doctors in training survey, with one green flag denoting performance in the top quartile of trusts nationally.

**Conclusion:**

The NTS can be analysed to create a plan of action with elements that trainers and trainees feel can improve their experience. Our model demonstrates the potential for using NTS data to plan quality improvement in training.

